# Supplementary material for: Heterologous Expression of Jatropha curcas Fatty Acyl-ACP Thioesterase A (JcFATA) and B (JcFATB) Affects Fatty Acid Accumulation and Promotes Plant Growth and Development in Arabidopsis
Source: Int J Mol Sci. 2022 Apr 11;23(8):4209. doi: 10.3390/ijms23084209 (PMC9029028; doi:10.3390/ijms23084209)
Supplement: Supplementary file 1 [file ijms-23-04209-s001.zip › ijms-1634570-supplementary.pdf]

Supplemental Materials

Heterologous Expression of *Jatropha curcas Fatty Acyl-ACP Thioesterase A* (*JcFATA*) and *B* (*JcFATB*) Affects Fatty Acid Accumulation and Promotes Plant Growth and Development in *Arabidopsis*

Supplemental Figures S1–S4 and Supplemental Tables S1–S6

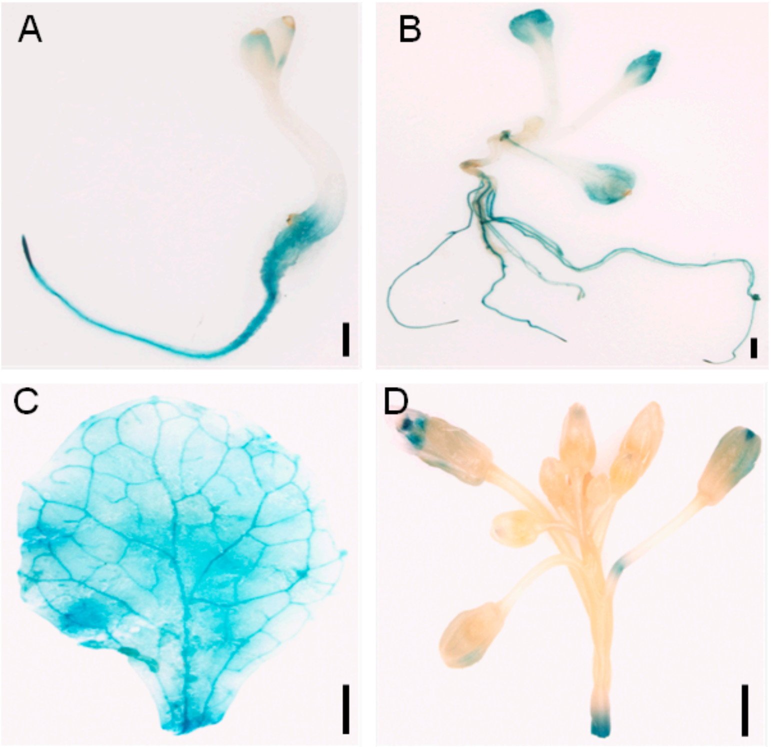

Figure S1. Expression patterns of *JcFATA* detected by GUS statining.
(A-B) *JcFATA*:*GUS* seedlings at 5- and 15-DAG; (C) A true leaf from 30-DAG *JcFATA*:*GUS* plant; (D) The top part of an inflorescence of the *JcFATA*:*GUS* plant. Scale bars = 1 mm. The experiment were conducted under the same conditaion as the *JcFATB*:*GUS* plants simultaneously.

```
AtFATA      ---------------------------------------------MLKLS
JcFATA      --------------------------------------------MMLKVP
AtFATB      MVATSATSSFFPVPSSSLDPNGKGNKIGSTNLAGLNSAPNS-GRMKVKPN
JcFATB      MVATAATSSFFPVPTSSADSKSTKIGSGSASLGGIKSKPASSGGLQVKAN

AtFATA      CNVTDHIHNLFSNSRRIFVPVHR------------QTRPISCFQLKKEPL
JcFATA      CTAPDQIQSLAPQCR--FLNQHRSS--------SHFTRRRANAVVYSSPV
AtFATB      AQAPPKINGKKVGLPGSVDIVRTDTETSSHPAPRTFINQLPDWSMLLAAI
JcFATB      AQAPPKINGSTVGYTTPVDSVKNEGDTPS-PPPRTFINQLPDWSMLLAAI

AtFATA      RAILSADHGNSSVRVADTVSGTSPADRLRFGRLMEDGFSYKEKFIVRSYE
JcFATA      AKISTQPVVSDGVTKVESG-CESLADRLRLGHLTEDGFSYKEKFIVRSYE
AtFATB      TTIFLAAEKQWMMLDWKPRRSDMLVDPFGIGRIVQDGLVFRQNFSIRSYE
JcFATB      TTIFLAAEKQWMMLDWKPRRPDMLIDPFGLGRIVQDGLVFRQNFSIRSYE

AtFATA      VGINKTATIETIANLLQEVACNHVQNVGFSTDGFATTLTMRKLHLIWVTA
JcFATA      VGINKTATVETIANFLQEVGCNHAQSVGFSTDGFATTPTMRKLHLIWVTA
AtFATB      IGADRSASIETVMNHLQETALNHVKTAGLLGDGFGSTPEMFKKNLIWVVT
JcFATB      IGADRTASIETLMNHLQETALNHVKTAGLLGEGFGSTPEMSKRNLIWVVT

AtFATA      RMHIEIYKYPAWSDVVEIETWCQSEGRIGTRRDWILKDCATGEVIGRATS
JcFATA      RMHIEIYKYPAWSDVVEIETWCQSEGRIGTRRDWILKDYATGQVIGRATS
AtFATB      RMQVVVDKYPTWGDVVEVDTWVSQSGKNGMRRDWLVRDCNTGETLTRASS
JcFATB      RMQVLVDRYPTWGDVVEVDTWVSASGKNGMRRDWLVRDSKTGETLTRASS

AtFATA      KWVMMNQDTRRLQRVTDEVRDEYLVFCPPEPRLAFPEENNSSLKKIPKLE
JcFATA      KWVMMNQDTRRLQKVTDDVRDEYLVFCPRELRLAFPEENNRSLKKISKLE
AtFATB      VWVMMNKLTRRLSKIPEEVRGEIEPYFVNSD--PVLAEDSRKLTKID--D
JcFATB      VWVMMNKLTRRLSKIPEEVRGEIEPYFLNSD--PIVDEDGRKLPKLD--D

AtFATA      DPAQYSMLGLKPRRADLDMNQHVNNVTYIGWVLESIPQEIIDTHELKVIT
JcFATA      DTTQYSKLGLVPRRADLDMNQHVNNVTYIGWVLESMPQEIIDTHELQTIT
AtFATB      KTADYVRSGLTPRWSDLDVNQHVNNVKYIGWILESAPVGIMERQKLKSMT
JcFATB      NTADYVCKGLTPRWSDLDVNQHVNNVKYIGWILESAPLPILESHELSSII
                               * *

AtFATA      LDYRRECQQDDIVDSLTTSETPNEVVSKLTGTNGSTTSSKREHNESHFLH
JcFATA      LDYRRECQHDDVVDSLTSAESLEGAGLDLHATNGSATAIAGEQDSRNFLH
AtFATB      LEYRRECGRDSVLQSLT-------------AVTGCDIGNLATAGDVECQH
JcFATB      MEYRRECGRDSVLQSLT-------------AVSGTGLGNLGNAGEIECQH
                  *

AtFATA      ILRLSENGQEINRGRTQWRKKSSR-------------
JcFATA      LLRFSSDGLEINRGRTEWRKKPSR-------------
AtFATB      LLRL-QDGAEVVRGRTEWSSKTPTTT--WGTAP----
JcFATB      LLRL-EEGAEIVRGRTAWRPKYRSNFGIMGQIPVESA
```

Figure S2. Sequence alignment of four FATA and FATB proteins of *A. thaliana* and *J. curcus*. The three predicted key enzyme activity sites were marked by “*” with red color. The sequence of AtFATA was for AtFATA1 (367 aa in length). Red boxes indicate the predicted transit peptide cleavage sites for JcFATA and JcFATB according to the previous study [34,35,42,43].

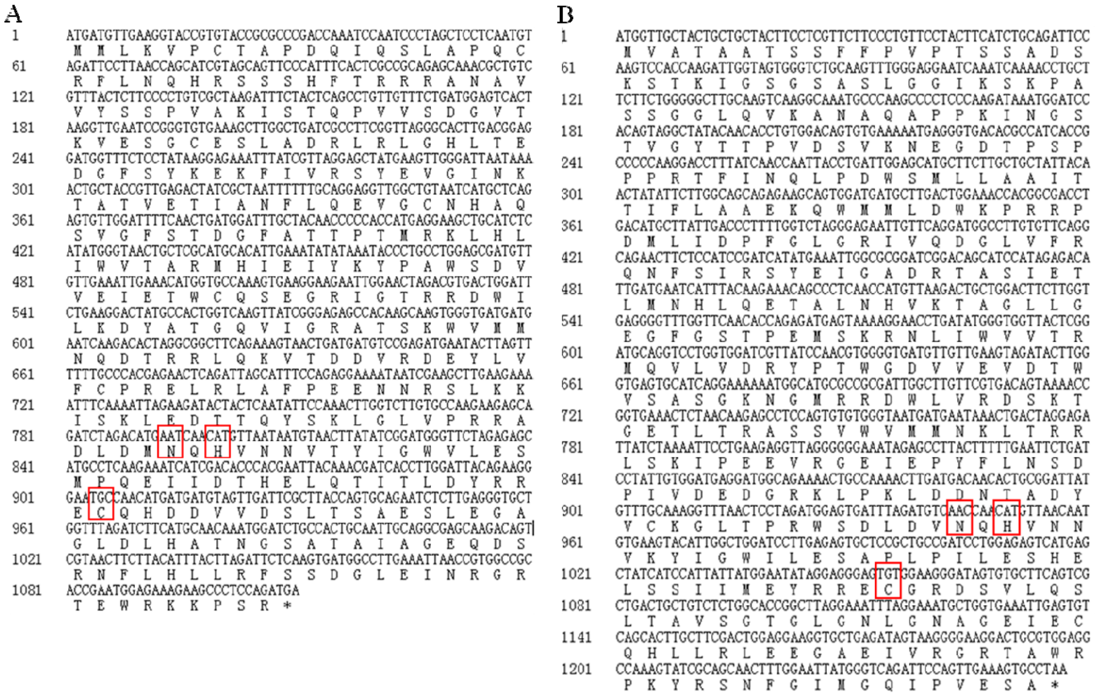

Figure S3. Amino acid sequences and cDNA sequences of *JcFATA* and *JcFATB*.

Amino acid sequence and cDNA sequence of *JcFATA* (A) and *JcFATB* (B). The amino acid residues circled with red boxes are the predicted enzyme activity sites for JcFATA and JcFATB.

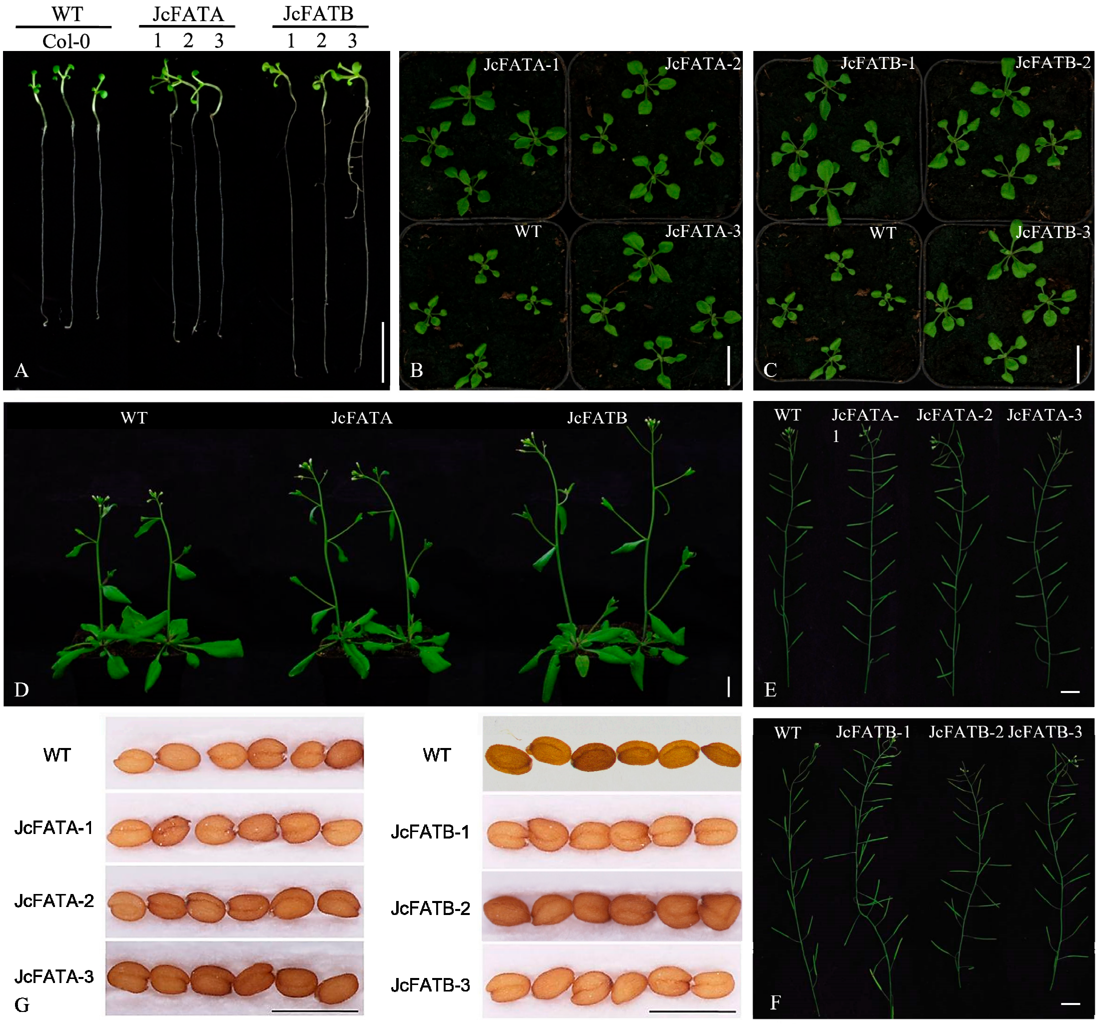

Figure S4. Phenotypes of the *A. thaliana* lines expressing *JcFATA* and *JcFATB* at different developmental stages.

(A) *A. thaliana* seedlings of the wild type and ectopic expression lines of *JcFATA* and *JcFATB* at 8-DAT (days after transplanting). (B-C) *A. thaliana* seedlings of the wild type and ectopic expression lines of *JcFATA* and *JcFATB* at 20-DAT. (D) *A. thaliana* plants of the wild type and ectopic expression lines of *JcFATA* and *JcFATB* at 28-DAT. (E-F) *A. thaliana* inflorescences and siliques of the wild type and ectopic expression lines of *JcFATA* and *JcFATB* at 50-DAT. (G) Mature seeds of *A. thaliana* lines of the wild type and ectopic expression lines of *JcFATA* and *JcFATB*. Scale bars indicated 1 cm in (A to F) and 1 mm in (G).

Table S1. The NCBI accession numbers for the mRNA and proteins used in this study.

Gene name	Accesssion number for CDS	Accesssion number for protein
JcFATA	EU267122.2 (1110 bp)	ABX82799.3 (369 aa)
JcFATB	EU106891.1 (1257 bp)	ABU96744.1 (418 aa)
AtFATA1	BT024746.1 (1104 bp)	ABD59084.1 (367 aa)
AtFATA2	NM_113415.4 (1089 bp)	NP_189147.1 (362 aa)
AtFATB	BT008505.1 (1239 bp)	AAP37864.1 (412 aa)
JcFAT-L1	JX966081.1 (1218 bp)	AGB56141.1 (405 aa)
JcFAT-L2	JX966082.1 (1047 bp)	AGB56142.1 (348 aa)
JcFAT-L3	JX966083.1 (1074 bp)	AGB56143.1 (357 aa)
JcActin7	XM_012232498.2 (1134 bp)	XP_012087888.1 (377 aa)
AtActin2	NM_112764.4 (1134 bp)	NP_188508 (377 aa)

Table S2. Fatty acid compositions and contents of mature seed storage lipids of *A. thaliana* transgenic lines expressing *JcFATA*.

Plant line	Fatty acid compositions and contents (ng/mg)									
	16:0	16:1	18:0	18:1	18:2	18:3	20:0	20:1	22:0	22:1
WT	5.36±1.13a	0.30±0.07b	2.92±0.76b	7.27±1.18b	19.65±1.19b	17.24±0.86b	1.02±0.23b	10.61±1.32a	0.23±0.20b	1.01±0.04b
JcFATA-1	7.23±1.45a	0.45±0.09a	5.16±1.05a	14.20±1.36a	32.63±4.06a	29.38±4.20a	1.38±0.19a	15.01±2.65a	0.35±0.07a	1.26±0.13a
JcFATA-2	6.68±0.64a	0.42±0.07ab	4.27±0.52a	12.67±1.99a	31.14±3.99a	27.28±3.17a	1.31±0.07ab	14.23±3.76a	0.30±0.06ab	1.22±0.09a
JcFATA-3	7.21±1.16a	0.45±0.06a	3.96±0.45a	12.31±2.05a	33.04±4.61a	26.51±3.91a	1.36±0.14a	12.73±2.56a	0.46±0.10a	1.28±0.12a

Values represent means ± SD (standard deviation), and 10 mg of dry seeds were used for each replicate and three replicates were assayed for each line. Data in the same column followed by different letters (a, b) are significantly different at $p \leq 5\%$ level as determined by Duncan's multiple range test.

Table S3. Fatty acid compositions and contents of mature seed storage lipids of *A. thaliana* transgenic lines expressing *JcFATB*.

Plant line	Fatty acid compositions and contents (ng/mg)									
	**16:0**	**16:1**	**18:0**	**18:1**	**18:2**	**18:3**	**20:0**	**20:1**	**22:0**	**22:1**
WT	5.36±1.13b	0.30±0.07a	2.92±0.76b	7.27±1.18b	19.65±1.19b	17.24±0.86b	1.02±0.23b	10.61±1.32a	0.23±0.20b	1.01±0.04a
JcFATB-1	11.13±1.92a	0.37±0.07a	7.15±1.36a	10.76±1.68a	24.49±3.29ab	23.65±2.54a	1.96±0.45a	10.88±1.82a	0.59±0.18a	0.61±0.17b
JcFATB-2	10.75±1.99a	0.34±0.09a	7.14±1.33a	9.93±1.65ab	23.16±2.18ab	21.86±2.61a	1.68±0.32a	10.75±1.39a	0.45±0.06ab	0.50±0.12b
JcFATB-3	9.86±2.73a	0.36±0.10a	6.53±2.97a	10.49±1.82a	25.26±3.71a	22.88±2.33a	1.71±0.27a	11.09±2.02a	0.62±0.22a	0.63±0.23b

Values represent means ± SD (standard deviation), and 10 mg of dry seeds were used for each replicate and three replicates were assayed for each line. Data in the same column followed by different letters (a, b) are significantly different at $p \leq 5\%$ level as determined by Duncan's multiple range test.

Table S4. Relative percentage of the target fatty acids in the seed storage lipids of the *A. thaliana* plants expressing *JcFATA* and *JcFATB*.

Fatty acids	Percentage（%）						
	**WT**	**JcFATA-1**	**JcFATA-2**	**JcFATA-3**	**JcFATB-1**	**JcFATB-2**	**JcFATB-3**
16:0	6.84±1.95ab	5.38±1.81b	5.41±0.90b	6.53±1.44ab	9.59±1.64ab	9.93±4.01a	8.47±2.31ab
16:1	0.37±0.06a	0.33±0.04a	0.35±0.13a	0.40±0.04a	0.31±0.05a	0.32±0.09a	0.31±0.08a
18:0	3.18±1.28c	3.78±0.99bc	3.47±0.82c	3.59±0.73c	6.07±1.18ab	6.75±1.09a	5.50±2.15abc
18:1	9.26±2.31b	10.32±0.73ab	13.52±3.98a	11.10±2.18ab	9.12±1.07b	9.39±1.38b	8.98±1.41b
18:2	24.69±0.89ab	23.71±2.42ab	25.36±5.76ab	29.65±4.03a	20.87±3.27b	21.93±1.67b	21.61±2.34b
18:3	21.75±2.25a	21.47±6.15a	22.54±6.35a	23.64±1.13a	20.14±2.52a	20.72±2.47a	19.56±0.64a
20:0	1.11±0.36abc	1.01±0.27c	1.08±0.24bc	1.22±0.16abc	1.67±0.44a	1.59±0.28ab	1.48±0.27abc
20:1	11.59±3.68a	10.65±1.59a	11.20±0.73a	11.37±1.82a	9.29±1.86a	10.16±1.05a	9.45±1.06a
22:0	0.34±0.31a	0.26±0.07a	0.25±0.09a	0.42±0.12a	0.50±0.17a	0.42±0.06a	0.53±0.17a
22:1	1.11±0.33a	0.93±0.20a	1.00±0.22a	1.16±0.17a	0.52±0.16b	0.47±0.10b	0.54±0.19b
SFAs	11.47±3.9	10.43±3.14	10.21±2.05	11.76±2.45	17.83±3.43	18.69±5.44	15.98±4.9
USFAs	68.77±9.52	67.41±11.13	73.97±17.17	77.32±9.37	60.25±8.93	62.99±6.76	60.45±5.72
Total	80.24±13.42	77.84±14.27	84.18±19.22	89.08±11.82	78.08±12.36	81.68±12.2	76.43±10.62

The data are means of three independent experiments, processed by the software SPSS 22.0 and conducted with Duncan multiple comparisons ($P \leq 0.05$). And

a significant difference was represented by different letters (a, b, and c). The total peak area of each measured fatty acid was calculated, and then the total peak area was subtracted from the peak area of ethyl decanoate (standard substance) to obtain the effective total peak area, and the percentage of the target fatty acids in seed oil was obtained by dividing the peak area of each component by the effective total peak area. Target fatty acids include 16:0, 16:1, 18:0, 18:1, 18:2, 18:3, 20:0, 20:1, 22:0, 22:1 as presented in Figure 5. SFAs: total percentage of the saturated fatty acids including 16:0, 18:0, 20:0, and 22:0. USFAs: total percentage of the unsaturated fatty acids including 16:1, 18:1, 18:2, 18:3, 20:1, and 22:1. Total: the total percentage of the target SFAs and USFAs.

Table S5 Ratios of the target fatty acids content in mature seeds of *Arabidopsis* to the standard substance ethyl decanoate detected by GC-MS.

Lines	Ratios
WT	7.59±0.34d
JcFATA-1	12.35±0.80a
JcFATA-2	12.08±0.92a
JcFATA-3	11.50±0.90ab
JcFATB-1	10.62±0.48bc
JcFATB-2	9.98±0.31c
JcFATB-3	10.35±1.19bc

The data are means of three independent experiments, processed by the software SPSS 22.0 and conducted with Duncan multiple comparisons ($P \leq 0.05$). And significant difference was represented by different letters (a, b, c, and d). Target fatty acids include C16:0, C16:1, C18:0, C18:1, C18:2, C18:3, C20:0, C20:1, C22:0, C22:1 as presented in Figure 5. The total peak area of target fatty acids was calculated, and then the total peak area was divided by the peak area of ethyl decanoate (standard substance) to obtain the ratio of target fatty acids in seed fatty acid accumulation in wild type and ectopic expression line of *Arabidopsis*.

Table S6. Primers used in this study.

Primer name	Sequence (from 5' to 3')	Purpose
JcFATA-F	aaaaGAATTCatgatgttgaaggtaccgtgt (*Eco*R I)	Used for *JcFATA* overexpression vector construction
JcFATA-R	aaaaACTAGTtcatctggagggcttctttct (*Spe* I)	
JcFATB-F	aaaaAAGCTTatggttgctactgctgctac (*Hin*d III)	Used for *JcFATB* overexpression vector construction
JcFATB-R	aaaaACTAGTttaggcactttcaactgga (*Spe* I)	
JcFATA-RTSF	actgatgatgtccgagatga	Used for semi-quantitative RT-PCR of *JcFATA*
JcFATA-RTSR	ctgcaattgcagtggcagat	
JcFATB-RTSF	cctattgtggatgaggatgg	Used for semi-quantitative RT-PCR of *JcFATB*
JcFATB-RTSR	ttcctccagtcgaagcaagt	
JcACT7-SF	ttcctgctcatagtcaagtgc	Used for *JcActin7* amplification by semi-quantitative RT-PCR
JcACT7-SR	gttgcccctgaggaacaccca	
AtACT2-F	aacccaaaggccaacagaga	Used for *AtActin2* amplification by semi-quantitative RT-PCR
AtACT2-R	gcatgaggaagagagaaacc	
JcFATB-GUS-F	aaaaGCATGCattgtgtcgcgtgttaggtg (*Sph* I)	Used for *JcFATB* GUS fusion expression vector construction
JcFATB-GUS-R	aaaaGGATCCccaaatgaaggtttgacacc (*Bam*H I)	

JcFATA-PGFP-F	aaaaGGATCCatgttgaaggtaccgtgtacc (*Bam*H I)	Used for *JcFATA* transient expression vector construction
JcFATA-PGFP-R	aaaaGAATTCtctggagggcttctttctcc (*Eco*R I)	
JcFATB-PGFP-F	aaaaAAGCTTatggttgctactgctgctac (*Hin*d III)	Used for *JcFATB* transient expression vector construction
JcFATB-PGFP-R	aaaaGTCGACggcactttcaactggaatc (*Sal* I)	
JcFATB-PC-F	aaaaGAGCTCatggttgctactgctgctac (*Sac* I)	Used for construction of prokaryotic expression vector of *JcFATB*
JcFATB-PC-R	aaaaAAGCTTttaggcactttcaactgga (*Hin*d III)	
JcFATA-PC-FM	ggattacagaagggaatTccaacatgatgatgtag	Used for construction of *JcFATA* site-directed mutagenesis vector
JcFATA-PC-RM	ctacatcatcatgttggAattcccttctgtaatcc	
JcFATB-PC-FM	ggaatataggagggagtTtggaagggatagtgtgc	Used for construction of *JcFATB* site-directed mutagenesis vector
JcFATB-PC-FM	gcacactatcccttccaAactccctcctatattcc	

Note: The six successive upper letters in some primer sequences indicate the digestion site in each primer used for vector construction. The upper letter in each of the last four primers indicates the mutant site compared with the wild type genes.
